# A study on the appropriate dose of rocuronium for intraoperative neuromonitoring in Da Vinci robot thyroid surgery: a randomized, double-blind, controlled trial

**DOI:** 10.3389/fendo.2023.1216546

**Published:** 2023-09-07

**Authors:** Jianning Lan, Qijian Huang, Jiansheng Su, Xuru Zhang, Liangcheng Zhang

**Affiliations:** ^1^ Department of Anesthesiology, Fujian Medical University Union Hospital, Fuzhou, China; ^2^ Department of Anesthesiology, Clinical Oncology School of Fujian Medical University, Fujian Cancer Hospital, Fuzhou, China

**Keywords:** intraoperative neuromonitoring(IONM), rocuronium, thyroidectomy, recurrent laryngeal nerve(RLN), Da Vinci robotic surgery

## Abstract

**Background:**

This study was to explore the effect of different doses of rocuronium bromide on neuromonitoring during Da Vinci robot thyroid surgery.

**Methods:**

This was a prospective, randomized, double-blind, controlled trial that included 189 patients who underwent Da Vinci robot thyroidectomy with intraoperative neuromonitoring(IONM). Patients were randomly divided into three groups and given three different doses of rocuronium (0.3mg/kg, 0.6mg/kg, 0.9mg/kg). Outcome measurements included IONM evoked potential, postoperative Voice Handicap Index-30(VHI-30), intraoperative body movement incidence rate, Cooper score, and hemodynamic changes during anesthesia induction.

Results: The difference in IONM evoked potentials at various time points between the three groups was not statistically significant (P>0.05). The difference in Cooper scores and intraoperative body movement incidence rate between 0.6 and 0.9mg/kg groups was statistically significant compared with the 0.3mg/kg group (both P<0.001). There was no statistically significant difference in VHI-30 score and hemodynamic changes during anesthesia induction among the three groups (both P>0.05).

**Conclusions:**

For patients undergoing Da Vinci robot thyroidectomy, a single dose of rocuronium at 0.6 and 0.9mg/kg during anesthesia induction can provide stable IONM evoked potential. Additionally, compared to 0.3 mg/kg, it can offer better tracheal intubation conditions and lower incidence of body movements during surgery. It is worth noting that the use of higher doses of rocuronium should be adjusted based on the duration of IONM and local practices.

## Introduction

In recent years, the incidence of thyroid-related diseases requiring surgical treatment has been gradually increasing (1). The operation space for thyroid and parathyroid surgery is small, and the anatomical protection of the recurrent laryngeal nerve (RLN) and parathyroid requires meticulous operation. The Da Vinci robotic surgical system has become an ideal auxiliary device for such surgeries due to its flexible operation and minimal damage (2, 3). Intraoperative neuromonitoring (IONM) can help reduce the incidence of RLN injury (4, 5). Neuromuscular blockers can provide good tracheal intubation conditions, improve surgical conditions, reduce surgical difficulty, and reduce iatrogenic injuries (6, 7).

However, studies have found that neuromuscular blockers can significantly affect IONM (8, 9). Currently, rocuronium is the most widely used neuromuscular blocker, and the recommended dose is based on open thyroid surgery (9–11). A lower dose of rocuronium (0.3 mg/kg, equivalent to 1 ED95) may be considered during anesthesia induction to accommodate intraoperative neuromonitoring (IONM) requirements. However, it’s important to acknowledge that this lower dosage not only has the potential to worsen intubation conditions but also carries the risk of increased patient movements, not only during tracheal intubation (11) but also throughout the first part of the intraoperative period. Inadequate neuromuscular blockade can result in intraoperative movement (12). In addition, during the robotic surgery process, patients need to be absolutely still because coughing or body movement can cause potential tissue damage and force the surgery to be terminated (13). At the same time, the time from the start of anesthesia to RLN monitoring in robotic surgery is significantly longer than that in open surgery (14). Therefore, based on these theoretical foundations, increasing the dose of neuromuscular blockers for robotic thyroid surgery within a certain range will not have a significant impact on IONM.

There are no reports on the optimal dose of rocuronium required for Da Vinci robotic thyroid surgery. Therefore, we designed a prospective randomized controlled trial to investigate the effect of different doses of rocuronium on RLN monitoring during Da Vinci robotic thyroid surgery. This study aims to determine the optimal dose of rocuronium to meet the needs of anesthesia and surgery.

## Methods

### Patient data

This study included patients who underwent scheduled Da Vinci robotic thyroidectomy at Fujian Medical University Affiliated Union Hospital from November 24, 2021, to November 30, 2022. All patients are operated on by the same surgeon. The study was approved by the Ethics Committee of Fujian Medical University Affiliated Union Hospital (No. 2021YF051-01) and followed the principles set forth in the Helsinki Declaration and its amendments. The study was registered with the Chinese Clinical Trial Registry (ChiCTR2100054279). All patients have given written informed consent and have been consecutively enrolled in the study. Inclusion criteria: 1. Mallampati grade I-II; 2. Age 18-60 years; 3. Body mass index (BMI) 18-28 kg/m2; 4. ASA grade I-II. Exclusion criteria: 1. Emergency surgery patients; 2. Patients with severe cardiovascular disease; 3. Liver and kidney dysfunction; 4. Suspected difficult airway; 5. Patients with preoperative voice hoarseness or RLN injury or tumor invasion; 6. Patients who changed the surgical method during the operation. Withdrawal criteria: 1. Patients who automatically request termination of the study; 2. Patients with unexpected difficult airways; 3. Patients with RLN injury during surgery; 4. Patients with serious complications during the perioperative period.

### Randomization and blinding

Random grouping: This study adopts a randomized double-blind controlled trial. The experimental subjects were randomly divided into three groups by anesthesiologist A who did not know the experimental plan using statistical software SPSS 26.0(IBM Corporation, USA): ROC1 group (1 ED95 group: 0.3mg/kg rocuronium), ROC2 group (2 ED95 group: 0.6mg/kg rocuronium), and ROC3 group (3 ED95 group: 0.9mg/kg rocuronium). And anesthesiologist A put the grouping and drug configuration information into opaque envelopes. Double-blind design: Before anesthesia induction, anesthesiologist A who did not know the experimental plan opened the random grouping envelope in the drug preparation room to determine the corresponding number of patients in each group, extracted the drug into a 10ml syringe and labeled the drug name, and handed it over to the surgical researcher in the operating room for intravenous injection preparation for patients. The anesthesiologist responsible for anesthesia operation, experimental data recording, postoperative follow-up, and the patients in the experiment were unaware of the specific grouping situation. Unblinding plan: If the patient suspects that the muscle relaxation effect during operation leads to the inability to IONM, unblind emergency will be performed by opening the random grouping envelope and administering 2-4mg/kg sugammadex sodium injection (Patheon Manufacturing Services LLC, USA) according to the dose of rocuronium.

### Anesthesia procedure

Half an hour before surgery, 0.5mg of pentobarbital sodium was injected intramuscularly. Patients were all fasted for 10 hours and prohibited from drinking for 6 hours. Radial artery puncture and catheterization were performed and a 22G venous catheter needle was placed in the superficial vein of the forearm and lactated Ringer’s solution was administered intravenously. After entering the operating room, electrocardiography (ECG), heart rate (HR), invasive blood pressure (IBP), pulse oxygen saturation (SpO2), bispectral index (BIS, Covidien, USA), and train of four (TOF, GE, USA) were connected to the monitor for monitoring. After resting in the supine position for 5 minutes, SBP, DBP, MAP, and HR were measured and recorded once. The anesthetic management was standardized for all patients, using BIS and TOF as a reference. Anesthetic induction medication: Sufentanil 0.4ug/kg, etomidate 0.4mg/kg. Then the experimental drugs were obtained according to the double-blind method. After the patient’s eyelash reflex disappeared, muscle relaxation monitoring was performed simultaneously, and the experimental dose of rocuronium(N.V.Organon, Netherlands) was injected. When TOF=0, a video laryngoscope was used for nerve-monitoring tracheal intubation and a nerve-monitoring tracheal tube was placed through the mouth under video laryngoscope assistance (male: ID7.0, female: ID6.0). The electrode of the nerve monitoring tracheal tube catheter was in close contact with both vocal cords, and the electrode position was confirmed by self-checking with the neuromonitoring during surgery. The nasopharyngeal temperature was monitored during the surgery, and body temperature was maintained between 36.0-36.5°C using a balanced maintenance anesthesia approach involving propofol at a rate of 5mg/kg/h, remifentanil at 0.08ug/kg/min, and sevoflurane at 0.7 MAC. BIS value was maintained within the range of 40-60. At the conclusion of the surgery, an intravenous injection of sufentanil was administered at a dose of 10ug and flurbiprofen at a dose of 50mg.

### IONM and surgical procedures

IONM was performed using the “Neuro Monitoring SystemTM” (NIM-Response^®^3.0, Medtronic, Minneapolis, USA). The same-side vagus nerve and RLN were stimulated to evaluate the corresponding vocal cord movement response. Monitoring system settings: threshold 100μV, stimulation current 3mA, amplitude-frequency 30Hz, 4 stimulations per second, and a stimulation period of 100μs. Before stimulating the RLN, the vagus nerve was stimulated routinely, and IONM evoked potential was measured at 90, 100, 110, and 120 minutes after the first identification of RLN and rocuronium use (definition: V1 signal represents the IONM signal produced by stimulating the vagus nerve before dissection, and R1 signal represents the first identified IONM signal when stimulating RLN (15)). Surgical procedures (16) and signal loss algorithms followed the guidelines of the International Neuro Monitoring Research Group (17, 18).

### Outcome measurements

After the patient entered the operating room, vital signs were monitored and baseline SBP, DBP, MAP, and HR (T1) were recorded. SBP, DBP, MAP, and HR were recorded at the first TOF=0 (T2), 30s after inflation of the tracheal tube cuff (T3), and 5min after inflation of the tracheal tube cuff (T4), The grading of tracheal intubation was also recorded according to the Cooper grading system (19). Good and excellent scores are considered clinically acceptable. The time from the completion of rocuronium injection to the start of surgery (Ta), the time from the start of surgery to the completion of subcutaneous tunnel establishment (Tb), the time of mechanical arm placement completion (Tc), and the start of laryngeal nerve monitoring time (Td) were also recorded. Patient’s body movements and IONM signal values (R1, R90, R100, R110, and R120) were recorded during the operation. Intraoperative body movement is defined as the patient’s spontaneous breathing recovery, limb movement, swallowing, or coughing during the operation. Follow-up was performed on postoperative days 1, 10, and 2 months, and the Voice Handicap Index-30 (VHI-30) was used to evaluate patients’ voice disorders. In this study, our main outcome measure was the amplitude of IONM evoked potential and secondary outcomes included VHI scores, intraoperative body movement, and hemodynamic changes during anesthesia induction.

### Sample size and statistical analysis

After obtaining ethical approval, we conducted a preliminary experiment to verify the feasibility of the experimental design and obtained IONM data to further calculate the sample size. It was anticipated that the mean values of IONM amplitude in the three groups would be 1283, 1203, and 1028, respectively. The corresponding standard deviations for each group were 295, 267, and 283. One-Way Analysis of Variance F-Tests were performed with α=0.05 and β=0.10. Using PASS 15 software, a sample size of N=102 was calculated, considering a potential loss-to-follow-up and refusal rate of 20%. Thus, a minimum of 129 samples (43 individuals per group) were deemed necessary for the final analysis.

SPSS 26.0 statistical software (IBM Corporation, USA) was used for analysis. For normally distributed measurement data, results were expressed as $\bar{x}\pm SD$ , for non-normally distributed data, median [quartile range] was used. Repeated-measures analysis of variance was employed for analyzing repeated measurement data, while categorical data were analyzed using one-way ANOVA, chi-square test, or rank-sum test as appropriate. Statistical significance was set at p<0.05 for all comparisons.

## Results

**Figure 1:**
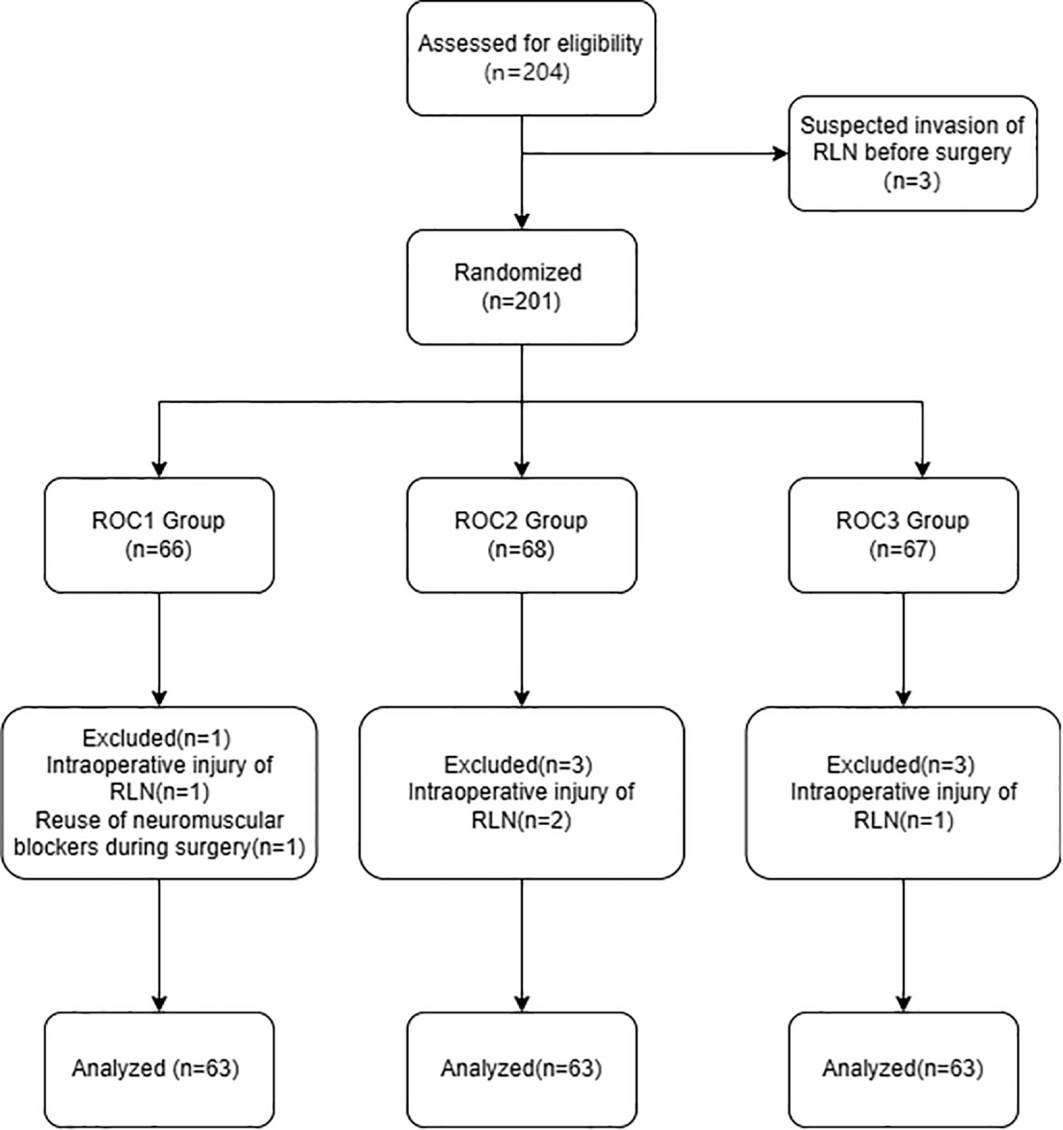
The flow diagram of patient selection in our study.

This study included a total of 189 patients who underwent elective thyroidectomy for papillary thyroid carcinoma. Patients were randomly assigned to three different doses of rocuronium test groups: ROC1 group (0.3mg/kg, n=63), ROC2 group (0.6mg/kg, n=63), and ROC3 group (0.9mg/kg, n=63). The flow chart is shown in **Figure 1**. There was no statistically significant difference in baseline characteristics such as age, weight, and height among the three groups of patients (**Table 1**). All patients underwent IONM evaluation, obtained complete IONM signal data successfully, and underwent relevant analysis.

**Table 1:** Demographic and perioperative characteristics.

	ROC1 (n=63)	ROC2 (n=63)	ROC3 (n=63)	p-value
Sex(M:F)	14:49	19:44	20:43	0.235a
Age (yr)	35.4 ± 7.9	37.9 ± 7.7	36.8 ± 8.2	0.562b
Weight (kg)	60.6 ± 10.4	62.2 ± 11.8	62.3 ± 12.2	0.180 b
Height (cm)	164.4 ± 7.9	165.7 ± 7.7	165.5 ± 8.2	0.769 b
BMI (kg/m2)	22.0[5]	22.0[4]	22.0[4]	0.895C

ROC1 group: 1x ED95 rocuronium (0.3mg/kg); ROC2 group: 2x ED95 rocuronium (0.6mg/kg); ROC3 group: 3x ED95 rocuronium (0.9mg/kg); BMI, body mass index; F, female; M, male; Data are expressed as $\bar{x}\pm SD$ or median [interquartile range]. a is analyzed by the chi-square test; b is analyzed by the independent sample T test; c is analyzed by the K-W test.

**Figure 2:**
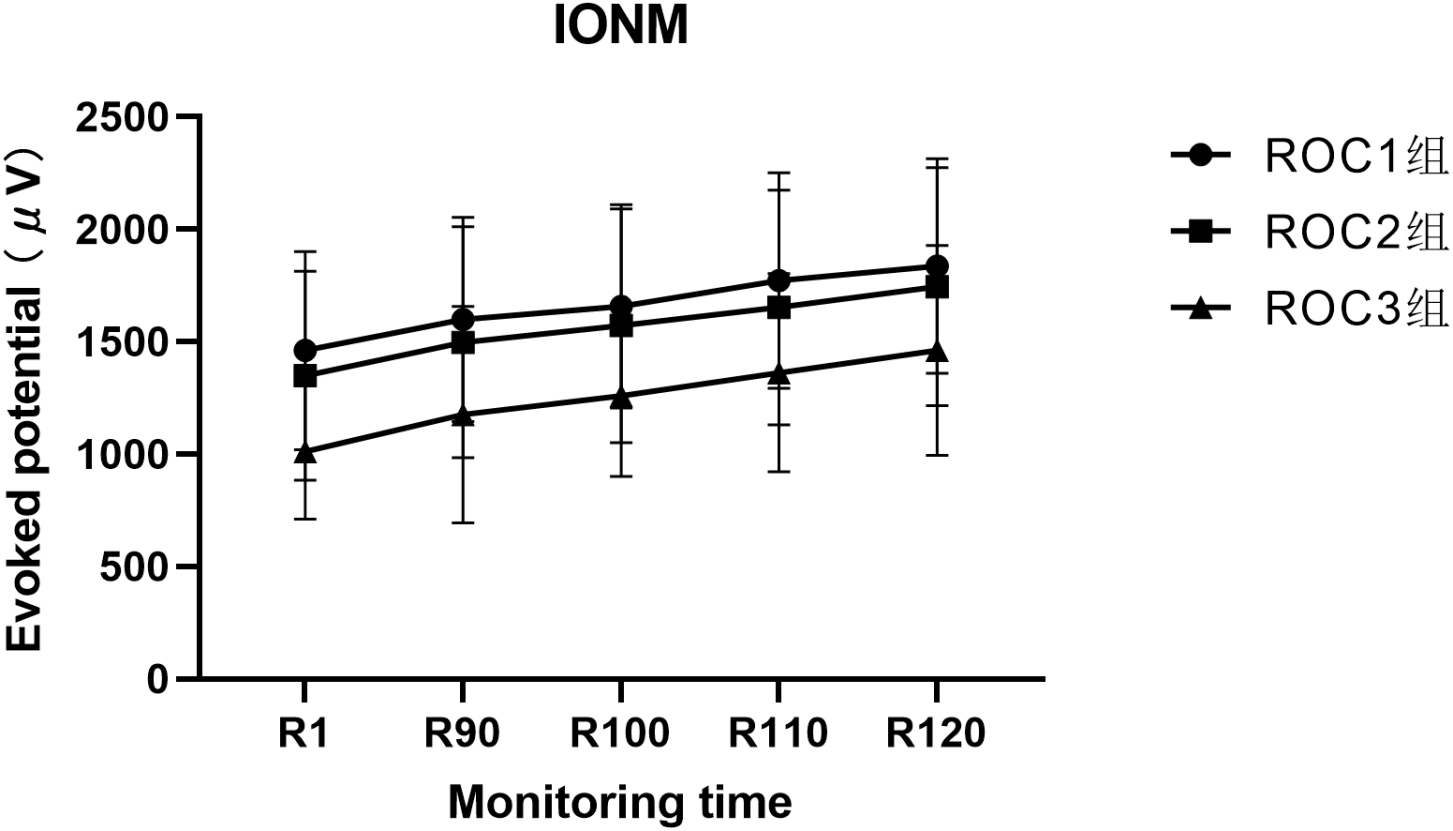
The Profile plot of IONM evoked potential at each monitoring time for three groups of patients.

There is no significant difference in IONM evoked potential among the three patient groups, but there is a significant difference at each time point. The IONM evoked potential is not the same at different time points, and the rate of change at different times in different groups is not the same. The pairwise comparison shows that the relationship between different times is R120>R110>R100>R90>R1 (**Table 2**; **Figure 2**).

**Table 2:** IONM evoked potential.

	ROC1 (n=63)	ROC2 (n=63)	ROC3 (n=63)
R1	1460.5 ± 440.8	1348.8 ± 465.1	1012.0 ± 300.4
R90	1598.7 ± 454.4	1497.7 ± 514.3	1176.1 ± 481.0
R100	1657.2 ± 451.5	1571.1 ± 519.4	1258.6 ± 358.5
R110	1771.8 ± 479.7	1652.7 ± 522.1	1362.3 ± 441.3
R120	1836.6 ± 477.7	1745.3 ± 529.4	1461.1 ± 467.2
F	180.5a	0.5b	15.6c
p-value	<0.001 a	0.692 b	<0.001 c

Unit: μV. The data are expressed as $\bar{x}\pm SD$ , and all p-values are obtained by repeated measurement variance analysis. a represents the within-group (time) effect, b represents the between-group (group) effect, and c represents the group × time effect. The uppercase letter 'F' represents the F-value or F-statistic.

In summary, there is no statistically significant difference in VHI-30 scores among the three patient groups. However, there is a statistically significant difference in VHI-30 scores at each time point, and there is no interaction between group and time. This means that the VHI-30 scores are the same at different time points and that the rate of change at different times in different groups is also the same. By comparing pairs, it can be seen that the relationship between different times is postoperative day 1 > postoperative day 10 > postoperative 2 months, and there is no statistically significant difference among the groups (**Table 3**).

**Table 3:** VHI scores.

	ROC1 (n=63)	ROC2 (n=63)	ROC3 (n=63)
Postoperative day 1 (day)	25.4 ± 22.1	26.0 ± 18.1	24.8 ± 21.7
10 (day)	7.7 ± 5.9	9.4 ± 6.8	8.7 ± 6.8
2 (month)	1.3 ± 1.3	1.2 ± 1.0	1.4 ± 1.2
F	254.760 a	0.179 b	0.098 c
p-value	<0.001 a	0.849 b	0.906 c

The data are expressed as x ®±SD, and all p-values are obtained by repeated measurement variance analysis. a represents the within-group (time) effect, b represents the between-group (group) effect, and c represents the group × time effect. The uppercase letter 'F' represents the F-value or F-statistic.

The statistical analysis revealed significant differences in SBP, DBP, and HR among the three groups at different time points. SBP exhibited a cross-effect between group and time, whereas DBP and HR had consistent rates of change at different time points. There was no significant difference in Ta, Tb, Tc, and Td time points between groups (all p>0.05) (For specific data, please refer to the supplementary materials). The results of Cooper scores and intraoperative body movements of the three groups of patients showed that all patients had successful tracheal intubation once. Compared with the ROC1 group, the intubation conditions of the ROC2 group and ROC3 group were better, and the difference was statistically significant (both p<0.001). Compared with the ROC1 group, the number of patients with intraoperative body movements in the ROC2 group and ROC3 group was less (both p<0.001), and there was no statistically significant difference between the ROC2 group and ROC3 group (p=0.285) (**Table 4**).

**Table 4:** COOPER score and intraoperative body movement.

	ROC1 a	ROC2 b	ROC3 b	T/χ2	p-value
Cooper grade				49.874	<0.001*
excellent	38	61	63		
good	21	2	0		
poor	4	0	0		
Incidence of body movement				28.525	<0.001**
Yes	29	12	4		
No	34	51	59		

*p-value is obtained by rank sum test, and there was no significant difference between groups with the same letter subscript. **p-value was obtained by chi-square test, and there was no significant difference between groups with the same letter superscripted.

## Discussion

With the advancement of diagnostic techniques for thyroid malignancies (20, 21), there is a rising trend in the number of patients requiring thyroid surgery. IONM technology has been widely utilized in thyroid surgery to real-time monitor the function of the recurrent laryngeal nerve. IONM technology has gained widespread use in thyroid surgery, enabling real-time monitoring of the recurrent laryngeal nerve’s function. For optimal protection of the RLN during thyroid surgery, IONM is strongly recommended (22). Therefore, it is crucial to examine the influence of muscle relaxant dosage on IONM. The muscle relaxation state must align with surgical requirements, minimize IONM impact, and avoid disrupting the surgical procedure. However, existing guidelines and studies lack information on the appropriate muscle relaxant dose for IONM in robotic thyroid surgery. Notably, the duration from anesthesia to IONM is longer in robotic procedures compared to open surgery (14), and laryngeal muscles tend to recover earliest from neuromuscular blockade (23), Moreover, in regular patients, neuromuscular blockers’ effect on IONM remains acceptable when the TOF count is ≥2 (24), Thus, in light of these factors, we initiated an RCT trial to explore the impact of varying rocuronium doses on IONM during robotic thyroid surgery. To our knowledge, this is the first RCT study to investigate the effect of different doses of rocuronium on IONM during robotic thyroid surgery.

Rocuronium, a commonly used neuromuscular blocker in clinical practice, is known for its rapid onset and moderate duration of action (25). The precise timing and quality of the IONM signal are of paramount importance when employing neuromuscular blockers in IONM. In our study, the average time from anesthesia induction to the first IONM signal in the three groups was recorded as 71.9 minutes, 70.9 minutes, and 71.6 minutes, respectively. Most patients experience a clinical duration of fewer than 60 minutes with a 0.9mg/kg dose of rocuronium (26). Overall, no statistically significant difference in the primary outcome measure, IONM peak value, was observed among the three groups. However, when compared to the 0.3mg/kg group, both group 0.6 and 0.9mg/kg groups demonstrated improved tracheal intubation conditions (p<0.001) and a lower incidence of intraoperative body movement (p<0.001). Intraoperative body movement, particularly involving the laryngeal muscles, can interfere with the accuracy of IONM monitoring and impact the surgeon’s interpretation of IONM signals (15). Our findings suggest that utilizing 0.6mg/kg and 0.9mg/kg mg/kg rocuronium during the anesthesia induction phase can yield stable IONM signals, improve tracheal intubation conditions, and reduce the occurrence of intraoperative body movement.

The VHI scores of the three groups of patients showed a downward trend over time, similar to that of Bea et al. (27). Given the similarities in surgical methods among all patients and the intact intraoperative IONM signals, it’s worth noting that the Cooper scores for all three patient groups were predominantly rated as “good” or higher. This might contribute to the absence of a significant difference in VHI scores among the groups. These findings are consistent with the results reported by Lee et al., reinforcing the notion that surgical method homogeneity and IONM signal integrity can influence VHI score parity (28).

At the T3 time point, we found that the hemodynamic changes in the ROC1 group were greater than those in the other two groups, but the difference was not statistically significant, which is consistent with the results of previous studies (29, 30). In the ROC1 group, the number of patients with intraoperative body movement increased significantly. Although the surgery was successfully performed after additional sedatives were added, one patient in the ROC1 group had a situation where the surgical instrument could not be inserted due to muscle tension. The ideal muscle relaxation for robotic thyroid surgery should meet the requirements of surgical operation, avoid affecting the operation, and not interfere with IONM. Studies have shown that appropriate neuromuscular blockade can provide appropriate neurophysiological monitoring conditions and improve the safety of anesthesia and surgery (24, 31). This indicates that our experiment has clinical significance. Overall, our study shows that a single dose of 0.6 and 0.9mg/kg rocuronium during anesthesia induction can provide stable IONM signals, reduce patient body movement, and have good tracheal intubation conditions. It is a more ideal dose in Da Vinci robotic thyroid surgery. However, it should be noted that the limitations of this study are that the sample size is relatively small and all patients are undergoing Da Vinci robot BABA approach thyroidectomy, and the results may be affected by specific surgical team experience and surgical methods. Despite the standardized anesthesia management, there was no recording and analysis of TOF and BIS values. Therefore, it is not possible to exclude the presence of significant unexamined confounding factors concerning patient movements in the three groups. Additionally, the routine use of video laryngoscopy for intubation could have influenced the results of this study, making intubation easier even at lower rocuronium dosages compared to direct laryngoscopy. At the same time, this study only compared the signals of recurrent laryngeal nerve, not V1, V2, R1, and R2 signals, which are different from the clinical practice specification, and the results obtained cannot be directly extended to the clinic. Additionally, most of the patients in this study are healthy young patients, and special populations (children, elderly) may have different research results due to differences in neuromuscular blocker metabolism. Therefore, caution is needed when applying the results of this study. In the future, more clinical trials and studies on the application of different doses of rocuronium in Da Vinci robotic thyroid surgery anesthesia in various approaches and special populations are still necessary to further ensure the safety and success rate of more patients.

## Conclusions

For patients undergoing Da Vinci robot thyroidectomy, a single dose of rocuronium at 0.6 and 0.9mg/kg during anesthesia induction can provide stable IONM evoked potential. Additionally, compared to 0.3 mg/kg, it can offer better tracheal intubation conditions and lower incidence of body movements during surgery. It is worth noting that the use of higher doses of rocuronium should be adjusted based on the duration of IONM and local practices.

## Data availability statement

The raw data supporting the conclusions of this article will be made available by the authors, without undue reservation.

## Ethics statement

The studies involving humans were approved by the Ethics Committee of Fujian Medical University Affiliated Union Hospital. The studies were conducted in accordance with the local legislation and institutional requirements. The participants provided their written informed consent to participate in this study. Written informed consent was obtained from the individual(s) for the publication of any potentially identifiable images or data included in this article.

## Author contributions

Authors statement JL: Conceptualization, Data Acquisition, Writing – original draft, Writing – review & editing. JS: Conceptualization, Methodology, Software, Visualization hang: Data Curation, Investigation QH: Supervision. LZ: Conceptualization, Methodology, Supervision, Visualization, Writing – original draft, Writing – review & editing. All authors contributed to the article and approved the submitted version.
