# Supplementary material for: A study on the appropriate dose of rocuronium for intraoperative neuromonitoring in Da Vinci robot thyroid surgery: a randomized, double-blind, controlled trial
Source: Front Endocrinol (Lausanne). 2023 Sep 7;14:1216546. doi: 10.3389/fendo.2023.1216546 (PMC10517056; doi:10.3389/fendo.2023.1216546)
Supplement: Supplementary file 1 [file Table_1.docx]

**Supplementary materials**

Table S1 Hemodynamic changes

|  | ROC1 | ROC2 | ROC3 | F | p |
| --- | --- | --- | --- | --- | --- |
|  | (n=63) | (n=63) | (n=63) |  |  |
| SBP（T1） | 144.4±16.1 | 141.3±18.7 | 141.7±17.0 | 412.412 a | ＜0.001 a |
| SBP（T2） | 111.7±13.4 | 112.5±15.5 | 111.8±11.4 | 2.521 b | 0.027 b |
| SBP（T3） | 152.3±24.5 | 144.1±22.7 | 146.5±21.8 | 1.489 c | 0.228 c |
| SBP（T4） | 113.4±14.1 | 110.9±11.4 | 113.4±14.3 |  |  |
| DBP（T1） | 78.0±8.8 | 77.2±9.7 | 75.9±8.8 | 315.808 a | ＜0.001 a |
| DBP（T2） | 64.0±8.4 | 65.2±10.1 | 64.5±7.3 | 2.304 b | 0.080 b |
| DBP（T3） | 87.9±14.3 | 84.6±14.4 | 84.1±11.0 | 0.244 c | 0.784 c |
| DBP（T4） | 64.6±7.5 | 65.4±7.8 | 66.5±8.6 |  |  |
| HR（T1） | 77.8±10.7 | 76.4±10.3 | 77.6±10.6 | 151.409 a | ＜0.001 a |
| HR（T2） | 72.2±13.2 | 74.1±11.8 | 74.4±11.3 | 3.186 b | 0.005 b |
| HR（T3） | 89.7±14.3 | 89.6±14.6 | 87.0±11.6 | 0.213 c | 0.808 c |
| HR（T4） | 72.4±12.1 | 76.1±11.8 | 77.1±10.6 |  |  |

Notes: The unit of blood pressure is mmHg. The unit of heart rate is beats per minute. The data are expressed as $\bar{x}\pm SD$, and all p-values obtained by repeated measurement variance analysis. a represents the within-group (time) effect, b represents the between-group (group) effect, and c represents the group × time effect.
