# Supplementary material for: A study on the appropriate dose of rocuronium for intraoperative neuromonitoring in Da Vinci robot thyroid surgery: a randomized, double-blind, controlled trial
Source: Front Endocrinol (Lausanne). 2023 Sep 7;14:1216546. doi: 10.3389/fendo.2023.1216546 (PMC10517056; doi:10.3389/fendo.2023.1216546)
Supplement: Supplementary file 2 [file Table_2.docx]

**Supplementary materials**

Table S2 Time at each point of anesthesia to nerve monitoring

|  | ROC1 | ROC2 | ROC3 | p-value |
| --- | --- | --- | --- | --- |
| Ta | 14.6±3.0 | 14.4±2.9 | 14.7±3.3 | 0.779 |
| Tb | 11.3±2.8 | 12.5±3.5 | 12.4±3.4 | 0.091 |
| Tc | 5.7±1.6 | 5.2±1.4 | 5.3±1.6 | 0.208 |
| Td | 71.9±6.6 | 70.9±5.8 | 71.6±5.8 | 0.644 |

Notes: Unit: min. The data are expressed as $\bar{x}\pm SD$, p-value is obtained by one-way analysis of variance (ANOVA).
